# A small vocal repertoire during the breeding season expresses complex behavioral motivations and individual signature in the common coot

**DOI:** 10.1186/s40850-021-00088-4

**Published:** 2021-09-02

**Authors:** Changjian Fu, Atul Kathait, Guangyi Lu, Xiang Li, Feng Li, Xiaoying Xing

**Affiliations:** 1 College of Wildlife and Protected Area, Northeast Forestry University, Hexing Road 26, Xiangfang District, Harbin, China; 2 School of Biosciences, Apeejay Stya University, Gurgaon, Haryana 122103 India; 3 Henan University of Urban Construction, Pingdingshan, 467036 China

**Keywords:** Behavioral motivation, Small call repertoire, Frequency and temporal parameters, Information coding, Rallidae, *Fulica atra*

## Abstract

**Background:**

Although acoustic communication plays an essential role in the social interactions of Rallidae, our knowledge of how Rallidae encode diverse types of information using simple vocalizations is limited. We recorded and examined the vocalizations of a common coot (*Fulica atra*) population during the breeding season to test the hypotheses that 1) different call types can be emitted under different behavioral contexts, and 2) variation in the vocal structure of a single call type may be influenced both by behavioral motivations and individual signature. We measured a total of 61 recordings of 30 adults while noting the behavioral activities in which individuals were engaged. We compared several acoustic parameters of the same call type emitted under different behavioral activities to determine how frequency and temporal parameters changed depending on behavioral motivations and individual differences.

**Results:**

We found that adult common coots had a small vocal repertoire, including 4 types of call, composed of a single syllable that was used during 9 types of behaviors. The 4 calls significantly differed in both frequency and temporal parameters and can be clearly distinguished by discriminant function analysis. Minimum frequency of fundamental frequency (F_0min_) and duration of syllable (T) contributed the most to acoustic divergence between calls. Call *a* was the most commonly used (in 8 of the 9 behaviors detected), and maximum frequency of fundamental frequency (F_0max_) and interval of syllables (TI) contributed the most to variation in call *a*. Duration of syllable (T) in a single call *a* can vary with different behavioral motivations after individual vocal signature being controlled.

**Conclusions:**

These results demonstrate that several call types of a small repertoire, and a single call with function-related changes in the temporal parameter in common coots could potentially indicate various behavioral motivations and individual signature. This study advances our knowledge of how Rallidae use “simple” vocal systems to express diverse motivations and provides new models for future studies on the role of vocalization in avian communication and behavior.

**Supplementary Information:**

The online version contains supplementary material available at 10.1186/s40850-021-00088-4.

## Background

Bird vocalizations are social signals that serve diverse functions, including mate attraction, territory defense, and social interaction with conspecifics or other species [1]. There are three basic mechanisms by which animals encode information in vocalizations [2]: song and syllable repertoire [3–5], frequency parameters, and temporal parameters [2, 6]. The first mechanism is used by open-ended learners of songbirds with extremely large song repertoires [4]; the second mechanism involves encoding information by simple changes in frequency and amplitude within syllables or notes [7, 8], which is a ubiquitous strategy used by vertebrates and many groups of invertebrates; and the third mechanism of encoding information is by changing the temporal distribution of vocalizations, such as temporal characteristics and delivery rate [9–11], to express behavioral motivations [6, 12]. Therefore, not only acoustic communication consisting of diverse types of syllables and elements expressing various meanings [13–16], the simple vocalizations, such as referential alarm calls can indicate categories of predators, or even predators’ behaviors [17–19]. For instance, noisy miner (*Manorina melanocephala*) emits ‘aerial’ alarm calls (high-frequency) to airborne raptor and produces ‘chur’ alarm calls (low-frequency and broad bandwidth) to terrestrial or perched raptor [20].

However, little is known about the referential functions of vocal behaviors in Rallidae, which appear to have more stereotyped and simpler vocalizations characterized by smaller repertoire sizes [2, 21]. Rallidae often gather in groups and have complex life history traits, such as breeding displays, alarm context and agonistic behaviors involving the broadcasting of loud calls during the breeding season, suggesting that the acoustic component of social interactions plays an important role in breeding interactions [6, 22–24]. Vocalizations of Rallidae are mainly “calls” that are uttered when they engage in courtship, mate attraction, territory guarding, and parent-offspring communication (e.g., the travel of newly hatched chicks led by their parents to feeding areas) [22]. Despite being subject to similar acoustic selective pressures and inhabiting the same habitats as other birds with complex vocalizations, how Rallidae express complex behavioral motivations using much simpler vocal types remains unclear [2].

A few recent studies have shown that Rallidae can use all three of these basic mechanisms including repertoire size, frequency and temporal modulation to encode information. Diverse types of information have been observed to be encoded in the vocal signals of rails [23, 25], crakes [2, 26] and corncrakes [6]. Modulation of the acoustic characteristics of the small vocal repertoire permits various types of information relating to breeding, species recognition and social signaling to be encoded. For example, information carried by the small repertoire of a single call type in petrels plays a role in social interactions, such as burrow defense and female mate choice, and acoustic parameters of energy quartiles, call duration, and syllable or phrase rate encode individual identity [22]. But these studies seldom considered to what extend the individual variations caused vocal structural differences in encoding behavioral motivations.

In this study, we used common coots to study how Rallidae code social interaction information such as mate attraction, territorial advertisement and individual signatures using single-syllable call types. Coots are good models to study vocal communication because it has a relatively small repertoire of innate calls, it normally breeds in wetlands with visibility often being restricted by dense vegetation, and vocalizations are known to play an important role in their social behavior [27]. They are highly territorial and produce loud advertisement calls consisting of a long series of identical, single-syllable notes throughout the daytime during the breeding season, which indicate the significance of vocal communications for successful breeding [28, 29]. Aggressive behaviors consisting of chasing or fighting with a long series of loud, identical, and single-syllable calls are frequently observed during the breeding season, suggesting that such simple calls encode multiple types of information such as physical quality (body size) or motivation and play an essential role in territory defense. Common coot parents produce sharp calls when leading nestlings to search for food, indicating a key role of vocalizations for parent-offspring communication [30].

Although previous work has described the vocal repertoires and displays of the American Coot (*F. americana*) [27], these preliminary studies were descriptive and did not use detailed acoustic analysis of the complete repertoire in a spectrogram to study the diverse behavioral contexts associated with their social interactions. Here, we provide a comprehensive overview of how the simple calls of the common coot encode diverse behavioral motivations by considering both vocal structure and the acoustic environment (i.e. natural factor in habitat such as vegetation) in which these vocalizations are produced. Specifically, we addressed 2 questions related to the functions of acoustic signaling: (1) Are different call types used in different behavioral contexts, such as aggression, courtship, foraging, or parent-nestling communication? and (2) Are acoustic parameters such as the frequency or temporal spectral domains modulated in ways that permit a single call type to express diverse behavioral motivations, and to what extent does individual signature contribute to the acoustic variations? We first identified and described various acoustic structures and their behavioral contexts under natural conditions to parse variation in the acoustic structure of calls according to different behavioral contexts during the breeding season. Second, we analyzed a general situation in which a specific type of vocalization was used to summarize how the common coot expresses information with a single call. Additionally, we evaluated different hypotheses for the relationship between the structure and presumed function of vocalizations among acoustic environments. By studying how a “simple repertoire” functions during breeding, we aimed to broaden our understanding of how diverse behavioral motivations are encoded in relatively simple systems [13].

## Results

### Different call types emitted under various behavioral contexts

**Fig. 1:**
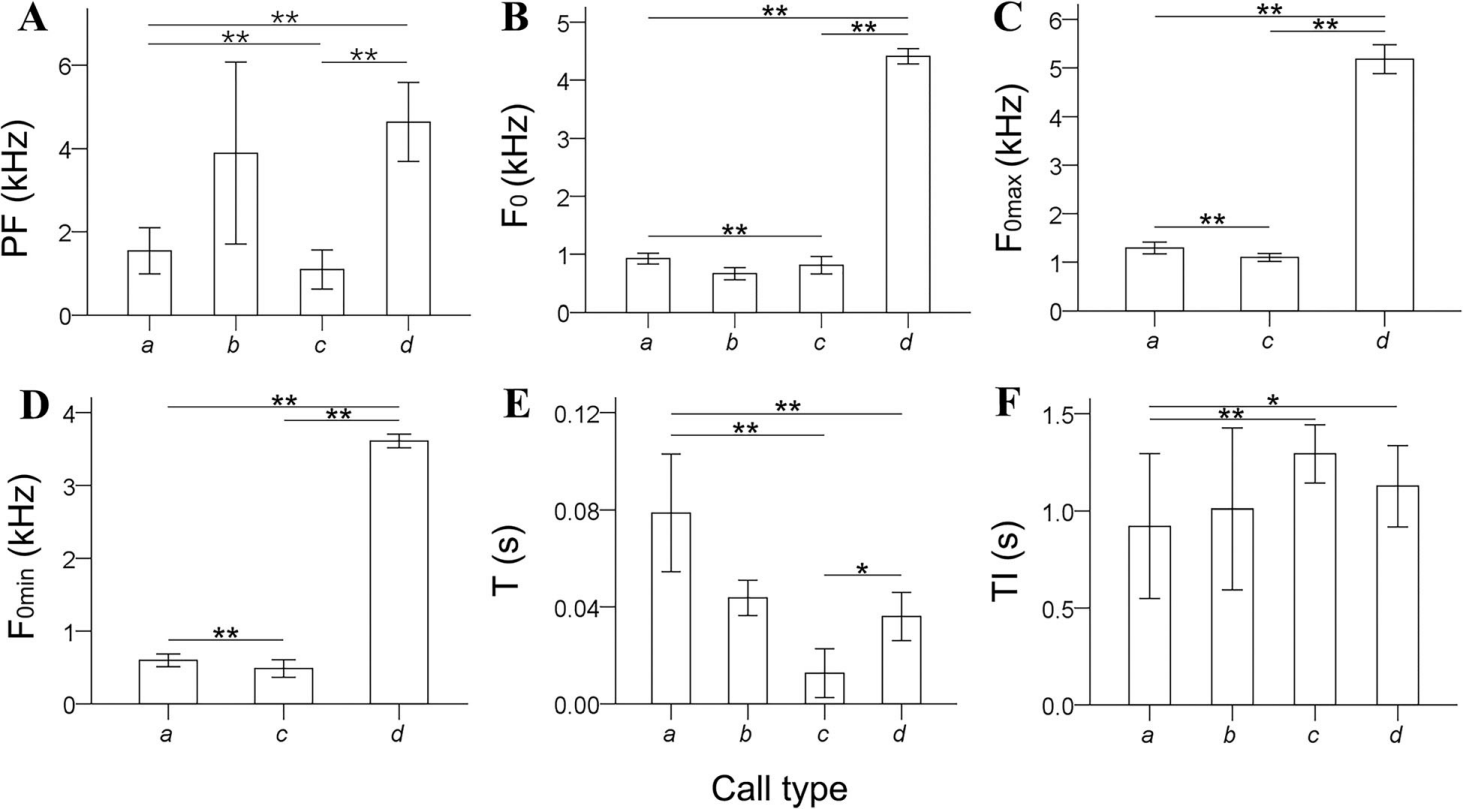
Acoustic comparisons among all 4 call types of adult common coot showing variation among different calls. Significant differences between any 2 types of calls are indicated by a line and * above the bar

ANOVA revealed that call types *a*, *b*, *c* and *d* were significantly different acoustically (Table 1, Fig. 1). Call *a* has the longest duration (Table 1; ANOVA, *a*-*c*: *F* = 72.311, *n* = 23 and 4, *P* < 0.001; *a*-*d*: *F* = 72.311, *n* = 23 and 4, *P* < 0.001) with the highest number of harmonics compared with the other 3 calls and was emitted during 8 of the behaviors observed in this study. Call *b* was produced during *leaving nest* or *communicating with nestlings*. Call *c* was the shortest in duration (Table 1; ANOVA, *a*-*c*: *F* = 72.311, *n* = 23 and 4, *P* < 0.001; *c*-*d*: *F* = 72.311, *n* = 4 and 4, *P* < 0.05) and had the longest intervals between syllables (Table 1; ANOVA, *a*-*c*: *F* = 14.294, *n* = 23 and 4, *P* < 0.001) with no harmonic; it was recorded during *back to nest* or *in the nest*. Call *d* had the highest maximum frequency (Table 1; ANOVA, *a*-*d*: *F* = 7923.200, *n* = 23 and 4, *P* < 0.001; *c*-*d*: *F* = 7923.200, *n* = 3 and 4, *P* < 0.001) and was only heard during *forage* on open water or *in the nest*.

**Table 1:** Results of one-way ANOVA showing significant differences among all 4 types of calls. *n* is the number of individuals. Paired comparisons between each of the 2 call types were subjected to least-significant difference tests

Call type	Peak frequency (PF, Hz)	Fundamental frequency (F_0_, Hz)	Maximum frequency of F_0_ (F_0max_, Hz)	Minimum frequency of F_0_ (F_0min_, Hz)	Duration of syllable (T, s)	Interval of syllables (TI, s)
*a*	1539 ± 555 (*n* = 23)	924 ± 96 (*n* = 23)	1291 ± 122 (*n* = 23)	597 ± 88 (*n* = 23)	0.080 ± 0.043 (*n* = 23)	0.921 ± 0.373 (*n* = 8)
*b*	3889 ± 2186 (*n* = 2)	663 ± 105 (*n* = 2)	–	–	0.044 ± 0.007 (*n* = 2)	1.010 ± 0.417 (*n* = 2)
*c*	1090 ± 471 (*n* = 4)	810 ± 151 (*n* = 4)	1096 ± 83 (*n* = 3)	485 ± 120 (*n* = 3)	0.013 ± 0.010 (*n* = 4)	1.293 ± 0.150 (*n* = 3)
*d*	4639 ± 948 (*n* = 4)	4411 ± 132 (*n* = 4)	5179 ± 297 (*n* = 4)	3610 ± 94 (*n* = 4)	0.036 ± 0.010 (*n* = 4)	1.127 ± 0.209 (*n* = 4)
ANOVA						
*F*	284.413	10,332.138	7923.200	9639.718	72.311	14.294
*P*	0.000**	0.000**	0.000**	0.000**	0.000**	0.000**
Paired comparisons						
*a-c*	0.000**	0.000**	0.000**	0.000**	0.000**	0.000**
*a-d*	0.000**	0.000**	0.000**	0.000**	0.000**	0.039*
*c-d*	0.000**	0.000**	0.000**	0.000**	0.035*	0.156

* *P* < 0.05 ** *P* < 0.01

### A single call type expresses multiple behavioral motivations

**Fig. 2:**
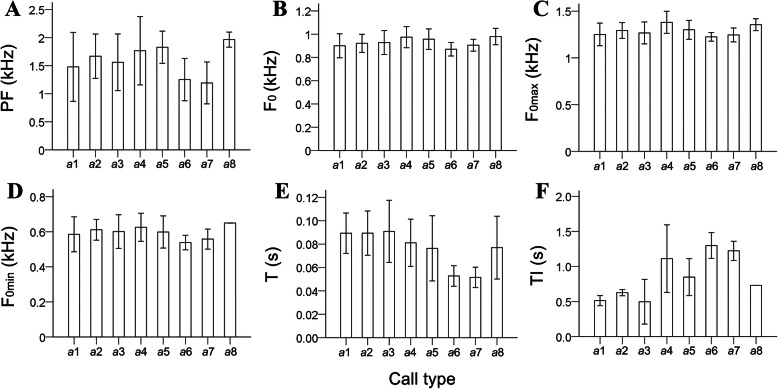
Acoustic variation among different call subtypes of *a* that were produced under 8 different behavioral contexts

The *a*1–*a*5 and *a*8 had higher frequency parameters (peak frequency, maximum frequency, and maximum/minimum frequency of F_0_), and longer durations with much faster syllable production than *a*6 and *a*7 (Fig. 2; Supplemental Table S1). According to LMM, only T had significant contribution (estimate ± *SE* = − 2.178 ± 0.912, *t* = − 2.387, *P* < 0.05) to classify the calls *a*1 and *a*3–*a*7 (Table 2). Means of frequency and temporal variables of calls *b*8, *b*9, *c*5, *c*6, *d*3 and *d*6 were shown on Table 3.

**Table 2:** The effect of PF, F_0_, F_0max_, F_0min_ and *t* on variations of call *a*1 and *a*3-*a*7 classified by different behavioral contexts

Variable	Estimate	*SE*	*t*	*P*
Intercept	4.245	0.865	4.908	0.000**
PF	−0.000	0.000	− 0.451	0.653
F_0_	0.001	0.001	1.100	0.272
F_0max_	−0.001	0.001	−1.865	0.063
F_0min_	0.001	0.001	0.751	0.453
T	−2.178	0.912	−2.387	0.018*

LMM using ‘lmer’ of the ‘lme4’ R package. Individual identity (ID) was included in the model as a random factor. Variance of ID = 2.358, *SE* = 1.536; variance of residual = 1.104, *SE* = 1.051. * *P* < 0.05 ** *P* < 0.01

**Table 3:** Means of acoustic parameters of calls *b*8, *b*9, *c*5, *c*6, *d*3 and *d*6, which are shown ± *SD*. *n* is the number of individuals

Call type	Behaviors	Peak frequency (PF, Hz)	Fundamental frequency (F_0_, Hz)	Maximum frequency of F_0_ (F_0max_, Hz)	Minimum frequency of F_0_ (F_0min_, Hz)	Duration of syllable (T, s)	Interval of syllables (TI, s)
*b*8	*leaving nest*	5553 ± 206 (*n* = 1)	727 ± 60 (*n* = 1)	–	–	0.044 ± 0.005 (*n* = 1)	1.842 ± 0.262 (*n* = 1)
*b*9	*communication with nestlings*	4240 ± 1938 (*n* = 1)	666 ± 128 (*n* = 1)	–	–	0.045 ± 0.007 (*n* = 1)	0.784 ± 0.040 (*n* = 1)
*c*5	*back to nest*	1265 ± 593 (*n* = 1)	985 ± 185 (*n* = 1)	–	–	0.012 ± 0.003 (*n* = 1)	1.111 ± 0.116 (*n* = 1)
*c*6	*in the nest*	1075 ± 464 (*n* = 3)	795 ± 140 (*n* = 3)	1096 ± 83 (*n* = 3)	485 ± 120 (*n* = 3)	0.013 ± 0.010 (*n* = 3)	1.326 ± 0.090 (*n* = 3)
*d*3	*forage*	4444 ± 159 (*n* = 3)	4444 ± 159 (*n* = 3)	5300 ± 287 (*n* = 3)	3567 ± 100 (*n* = 3)	0.034 ± 0.007 (*n* = 3)	1.090 ± 0.224 (*n* = 3)
*d*6	*in the nest*	4833 ± 1341 (*n* = 1)	4378 ± 97 (*n* = 1)	5058 ± 267 (*n* = 1)	3653 ± 68 (*n* = 1)	0.038 ± 0.012 (*n* = 1)	1.149 ± 0.116 (*n* = 1)

## Discussion

Rallidae have a simple vocal apparatus, and their simple syringeal anatomy is thought to constrain their vocal complexity and limit the diversification of call types within the vocal repertoire of Rallidae [31]. Nevertheless, they vocalize extensively with their small repertoires, and these vocalizations have important functions during breeding [2]. Our study supported these ideas, as only 4 different call types (*a*, *b*, *c*, and *d*) were recorded, all of which consisted of a long series of repeated single-syllable sounds under 9 different behaviors.

Despite a small repertoire of vocalizations, the common coot expressed diverse behavioral motivations. Specifically, the common coot modified the vocal structures of their simple acoustic systems in 3 ways. First, the common coot producing acoustically different call types that were clearly distinguished by DFA analysis in different behavioral contexts, and the minimum frequency of fundamental frequency (F_0min_) and duration of syllable (T) contributed the most to the acoustic divergence between call types. Call *c* had the shortest duration and was produced during *in the nest* and *back to nest*. Previous studies have shown that North American rails have high-frequency alarm calls that are characterized by short pulses, and the note duration of the alarm call of the king rail (*Rallus elegans*) is short, making it difficult for predators to detect [25, 32]. Because short notes are superior for avoiding detection by predators and can enrich information relating to direction and distance [33], the short *c* is often favored during parental interactions in common coots during *in the nest* or *back to nest*. common coots emitted call *d* when they were foraging for food on the water surface or in the nest; *d* had a significantly higher frequency compared with the other 3 calls. Because the location of vocalization producers can be easily detected by signal receivers through high-frequency calls [13, 19], *d* may be used to determine the location of mates and be used as a general contact call. Call *b* is the only call that we recorded for parent-offspring communication, which had the lowest fundamental frequency (F_0_) among all call types. According to the acoustic adaptation hypothesis, dense habitats favor the use of calls with lower frequencies, as low-frequency calls experience less acoustic degradation in dense habitats compared with high-frequency calls [34, 35]. Call *b* was used in the dense reeds and would thus be advantageous for its lower frequency duration transmission. Therefore, the behavioral contexts and the acoustic environment in which the call is produced both drive the vocal structures of common coot calls. Their relative, the American Coot has been shown to have similar ways of containing information, in which different call types are used for individual recognition, courtship, and alarm signals during nest/territory defense and communication between mates and parent-offspring [28].

common coots can also send information by changing the frequency and temporal parameters within a single call type. Call *a* was the most commonly used and was emitted in 8 of the 9 behaviors that were noted. Except *a*5 and *a*8 (which have few recordings, Supplemental Table S2), *a*1–*a*7 were correctly classified in the DFA analysis, and the maximum frequency of the fundamental frequency (F_0max_) and interval of syllables (TI) contributed the most to the classification. To modify frequency, common coots used *a*4, which had the highest F_0max_ during *chase and fight* with intruders, and used *a*6 and *a*7, which had the lowest F_0max_, during *in the nest* and *searching nest materials* on open water. Increases in frequency have been observed during the arousal of many vertebrates, including birds and mammals, as a way of expressing urgency [36–39]. The results of our study support this idea given that intruders are the main threat to breeding adults compared with contact with mates while in the nest or while searching for nest materials. Call *b* was produced when individuals leave the nest and during parent-offspring communication, and its much lower fundamental frequency (F_0_) when parents call to their chicks may represent an adaptation to dense habitat, as the use of low-frequency calls by adults to contact chicks is favored in complex acoustic environments [13].

Temporal distributions also enrich the ways by which common coots can express behavioral motivations. The *a*6 and *a*7 had the longest TI, which makes sense given that interactions *in the nest* and *searching for nest materials* on open water are generally some of more peaceful and slower activities that common coots engage in. Under the more urgent behavioral contexts of *a*1–*a*4 (*courtship*, *copulation*, *forage*, and *chase and fight*, respectively), TI is shorter, and thus *a*1–*a*4 are much faster. This finding suggests that common coots encode urgent situations by decreasing the temporal intervals between syllables and thus increasing the speed of syllable output. Call *d* emitted during *forage* had a significantly shorter TI than when call *d* was emitted during *in the nest*. Thus, we inferred that the former *d* functions as a contact call for mates and/or as territorial advertisement, both of which are activities that have a greater sense of urgency compared with activities while in the nest. This temporal modification depending on the degree of behavioral urgency has also been observed in many other animal taxa, including mammals [40] and songbirds [6, 41] but has only been documented in a few cases in Rallidae. For example, the spotted crake (*Porzana porzana*) lengthened their between-call intervals as an aggressive motivation [2]. Corncrakes (*Crex crex*) calls consist of 2 syllables separated by 2 intervals (I1 and I2); although I1 is generally similar to I2, males can produce calls that have a longer I2 than I1, which encodes information on the aggressive motivation to other males. That is, specific information can be encoded by the temporal pattern [6, 41].

Vocal individuality also contributes to acoustic parameters divergence [42–44]. Nevertheless, although vocal variation of call *a* among individuals was considered in our study (LMM), a parameter, Duration of syllable (T), was still differed significantly in different behavioral contexts, which indicates T is specifically used for expressing distinct behavioral motivation.

In this study, we classified these call types and subtypes by mainly acoustic traits analyses and spectrogram measurements, there was more difference among call types *a*, *b*, *c* and *d* than subtypes of *a* calls, and in visual, spectrogram subtypes of *a* was similar. However, some studies indicated that even extremely similar vocalizations were classified into different call types because they were produced in different behavioral contexts and encode contrasting function, for instance, the surprisingly similar hawk and mobbing alarm calls of superb fairy-wrens (*Malurus cyaneus*) [18] and aggressive and affiliative trill of Java sparrow (*Lonchura oryzivora*) [45]. Thus, the common methods we used for classified call types maybe not applicable to birds of Rallidae with simple calls, and call type classification should focus on not only acoustic parameters’ differences, but certain function or behavior context. Playback experiments are needed to test the function and classification of these call types further, with considering how sexes or individual differences lead to vocal variations in the future [46]. A playback experiment simulating territorial intrusion in the spotted crake reported that males can lengthen their between-call intervals to show aggressive motivation [2]. Finally, according to LMM, we found that both individuality and behavioral contexts contribute to variation of acoustic traits of different call types in common coots.

However, there are obvious limitations and some conclusions might surpass what the limited sample size and methods design can reach in our study. First, this is a more descriptive study which attempts to explore the relationship between behavioral context and acoustic parameters, and it’s restricted to only spectrogram analysis and behavioral observation without testing the responses of signal receivers. Second, the sample sizes of call *b*, *c*, *d* and some subtypes of call *a* such as *a*2 and *a*8 are critically low with a few individuals (only one in some cases), which hindered analyzing divergent functions of different call types. Third, the differences of acoustic parameters between male and female common coots that may contributed to the acoustic variation are not tested (but a LMM was conducted) in this study because the sex of each individual cannot be certain through morphology in field. Therefore, further experiments such as call manipulation or playback experiments are needed to conduct to shed light on the specific information encoding mechanisms of Rallidae in future.

## Conclusions

In sum, we provided the first detailed spectral analysis of common coot vocalizations, which indicated that common coots produce a few vocal types that containing various types of information under different behavioral contexts. The findings of this study on common coot, a member of the Rallidae, support the results of recent studies suggesting that even considering vocal individual signature, the vocal repertoire, acoustic structure, and temporal distributions of sounds provide three basic mechanisms by which vocalizations can encode information in species of Rallidae [2, 6]. This study also broadens our perspective on how birds emit complex functions using relatively simple acoustic signals, thereby increasing our understanding of the origin and evolution of small vocal repertoires. Our detailed spectrogram analysis of common coot vocalizations provides a foundation for future playback experiments to determine how subtle changes in calls modulate the information that the calls contain. Similar to the American coot, the vocalizations of the common coot play an important role in social behaviors [27, 28]; thus, anthropogenic sources of noise should be mitigated near the breeding areas of common coots to avoid disturbing their reproductive activities [47].

## Methods

### Recording protocol and behavioral contexts definition

In this study, we studied a population of common coot at Anbanghe National Reserve in Heilongjiang, China (46.8853°N–47.0650°N, 131.1033°E–131.5400°E). Common coots are strongly territorial and produce loud, brief, and sharp sounds all day during the breeding season, and intruders are expelled from territories immediately upon their entry. The stability of their territories thus permitted many individuals to be recorded while ensuring that different individuals were discriminated and identified. Recordings taken > 100 m apart were assumed to be different individuals based on estimated territory size and individually marked with number (see also Supplemental Table S2). The nest sites of common coots can be approached closely to make high-quality vocal recordings through an artificial corridor for tourists within the national reserve, thus we opportunistically recorded common coots who produce calls within an estimated distance of 10–30 m from focal birds along the artificial corridor between 05:00–10:00 h and 13:00–17:00 h from April to June 2008. Vocalizations of breeding adults were recorded using Portable Recorder (Lotoo L-200, Beijing, China) and a Directional Microphone (ΛZDEN SGM 1X, Tokyo, Japan) held approximately 1.5 m high on hands of the researcher. The duration of each recording did not exceed 2 min (except *b*9 with 5′47″) and were made at 16 bit resolution and sampling rate of 22.05 kHz (for calls *a* and *c*) or 44.1 kHz (for calls *b* and *d*), which have been demonstrated previously to be sufficient for the extraction of the acoustic parameters we measured [48]. For behavioral observation, we observed and defined certain behavior of common coot in breeding season within an estimated distance of 10–30 m from focal birds (the same as call recorded, which was synchronized with behavioral observation) and recorded these behaviors using a camera (Panasonic DMC-FZ18GK, Osaka, Japan) to ensure that the behaviors displayed while target call types were broadcast were also noted. In total, we noted 9 types of behaviors that were displayed when common coots produced calls (Table 4).

**Table 4:** Behavioral contexts list, description of behaviors and call types produced under these contexts

No.	Behavioral contexts	Description
1	*courtship*	The male chases the female on the water surface before copulation.
2	*copulation*	The male stands on the female’s back to copulate while pecking its head and producing loud and rapid calls on the water surface or in the nest.
3	*forage*	The common coot makes continuous calls when foraging for food in open water.
4	*chase and fight*	The common coot fights and chases away other conspecifics, especially during the nest-building period, often involving pecking with the bill, kicking with the feet, and the broadcasting of hurried and loud calls.
5	*back to nest*	2 different types of call were recorded when common coots swam into the dense reeds and back to the nest.
6	*in the nest*	The common coots can make 3 different weak calls while they are preening feathers or building nests in the reeds.
7	*searching nest materials*	The common coots make the most commonly heard call type gently when they are searching and picking up dried or died grass stems to build nests on the water surface.
8	*leaving nest*	2 different calls when common coots leave nests and swim out of the reeds.
9	*communication with nestlings*	A specific call type which was most rarely recorded produced by the adult common coots to seek or interact with their nestlings.

### Vocal analysis

**Fig. 3:**
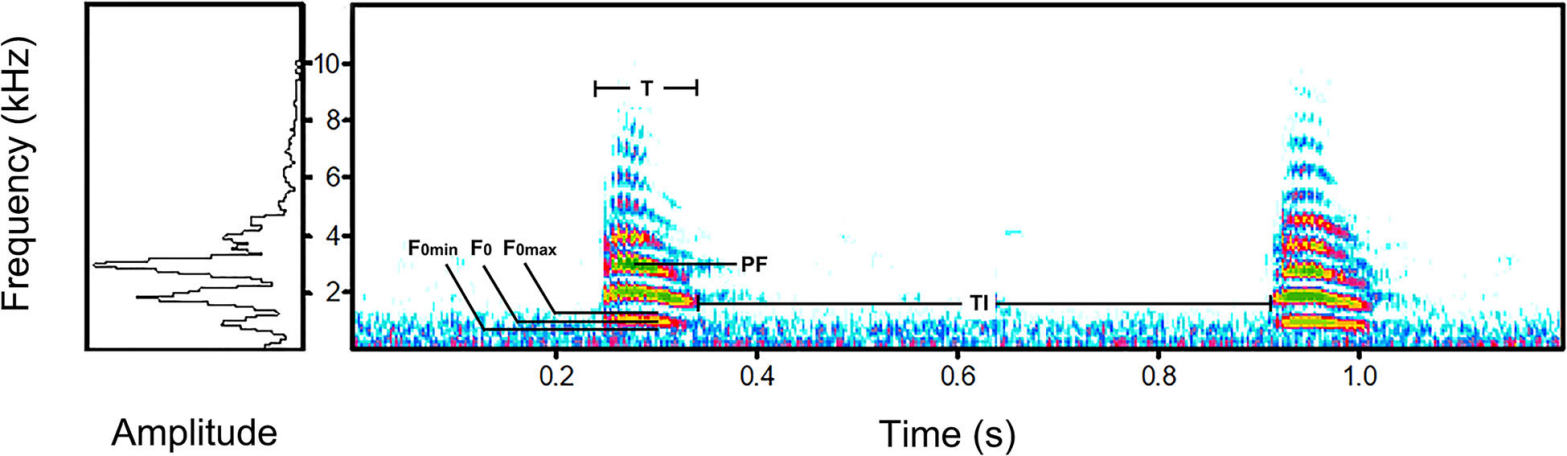
Sonogram of a common coot call to demonstrate how parameters were measured for each syllable. The parameters included peak frequency (PF), fundamental frequency (F_0_), maximum frequency of fundamental frequency (F_0max_), minimum frequency of fundamental frequency (F_0min_), duration of syllable (T), and interval of syllables (TI). Amplitude spectra of the left syllable show high energy in the third harmonic (PF)

**Fig. 4:**
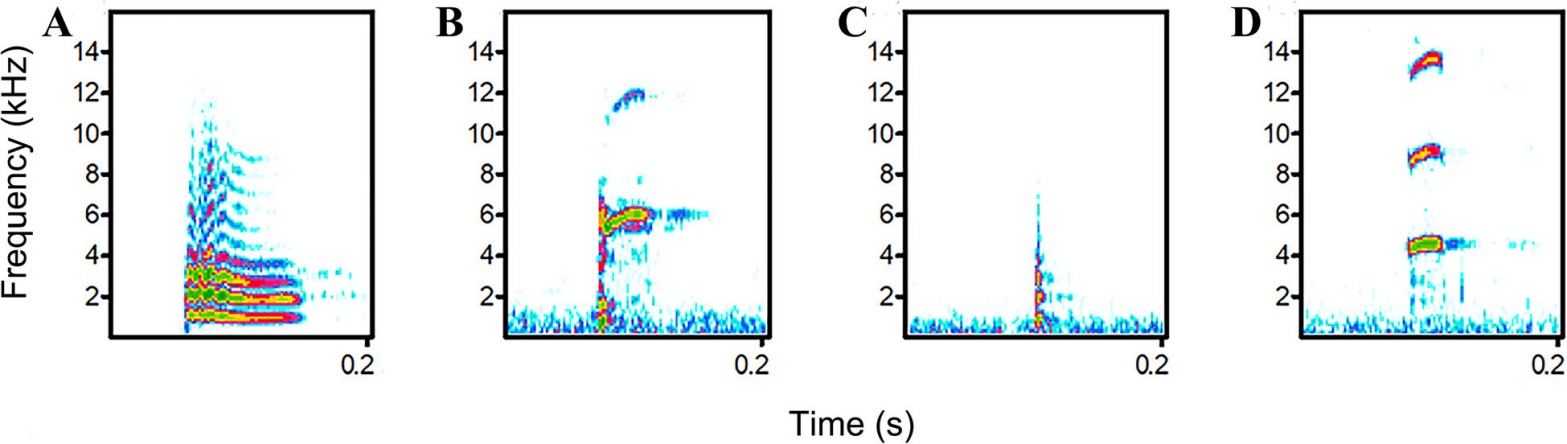
Four types of calls—*a*, *b*, *c*, and *d*—of adult common coots in the breeding season are shown in (A)—(D) respectively

The vocalizations were analyzed in Avisoft-SASLab Pro 4.52 (Avisoft Bioacoustics Inc., Berlin, Germany); the waveforms and spectrograms for analyses were created using FFT-length 512 points, Hamming window, frame 50%, and overlap 75%. WAV sound files. Calls that were undisturbed by other sounds (e.g., man-made noise, vocalizations of anurans or other bird species in the habitat of common coot) and possessed a high signal-to-noise ratio (S/N) were selected for analysis. We classified the vocalizations into different call types (i.e. syllables) according to spectrogram characteristics and then we classified these call types further by different behavioral contexts in which different call types were used, which simple elements separated by noticeable time intervals on the spectrograms are defined as syllables [49], i.e. calls for common coots. In order to do so, we measured 6 variables: (1) peak frequency (PF), (2) fundamental frequency (F_0_), (3) maximum frequency of fundamental frequency (F_0max_), (4) minimum frequency of fundamental frequency (F_0min_), (5) duration of syllable (T), and (6) interval of syllables (TI, Table 5; Fig. 3), and we chose these variables following some previous similar studies [2, 25, 50, 51]. We identified 4 different types of calls consisting of repeated, single-syllable calls from 61 recordings of 30 breeding adults (see also Supplemental Table S2), which were called *a* (46 recordings including 517 calls from 23 individuals), *b* (2 recordings; 215 calls; 2 individuals), *c* (8 recordings; 59 calls; 4 individuals), and *d* (5 recordings; 18 calls; 4 individuals), 809 calls in total. The 4 types of calls are easily distinguishable through visual observation in the spectrograms (Fig. 4). Call *a* was emitted under 8 different behaviors and was thus the most frequently used among the 4 call types. The numbers 1–8 were used to refer to the different behavioral contexts where *a* is emitted (*a*1, *a*2, *a*3, *a*4, *a*5, *a*6, *a*7, and *a*8, Supplemental Table S1, Fig. S1). The *a*2 and *a*8 were excluded from statistical analyses because only a few syllables from one individual were recorded for each of these behavioral contexts. The calls *b*, *c*, and *d* were only emitted under 2 behaviors. The aforementioned numbering was also applied to *b*, *c*, and *d* (*b*8, *b*9, *c*5, *c*6, *d*3, and *d*6).

**Table 5:** Parameters and definition of various vocal parameters measured for each call types

Parameters	Definition
Harmonic	A series of musical tones with several times frequencies of F_0_.
PF	The frequency of maximum energy in the power spectrum of a target syllable.
F_0_	The frequency of the first harmonic peak in the power spectrum of a target syllable.
F_0max_	Highest frequency of the first harmonic of a target syllable.
F_0min_	Lowest frequency of the first harmonic of a target syllable.
T	Total duration of a target syllable.
TI	Duration between a syllable and a next one.

### Statistical analysis

**Fig. 5:**
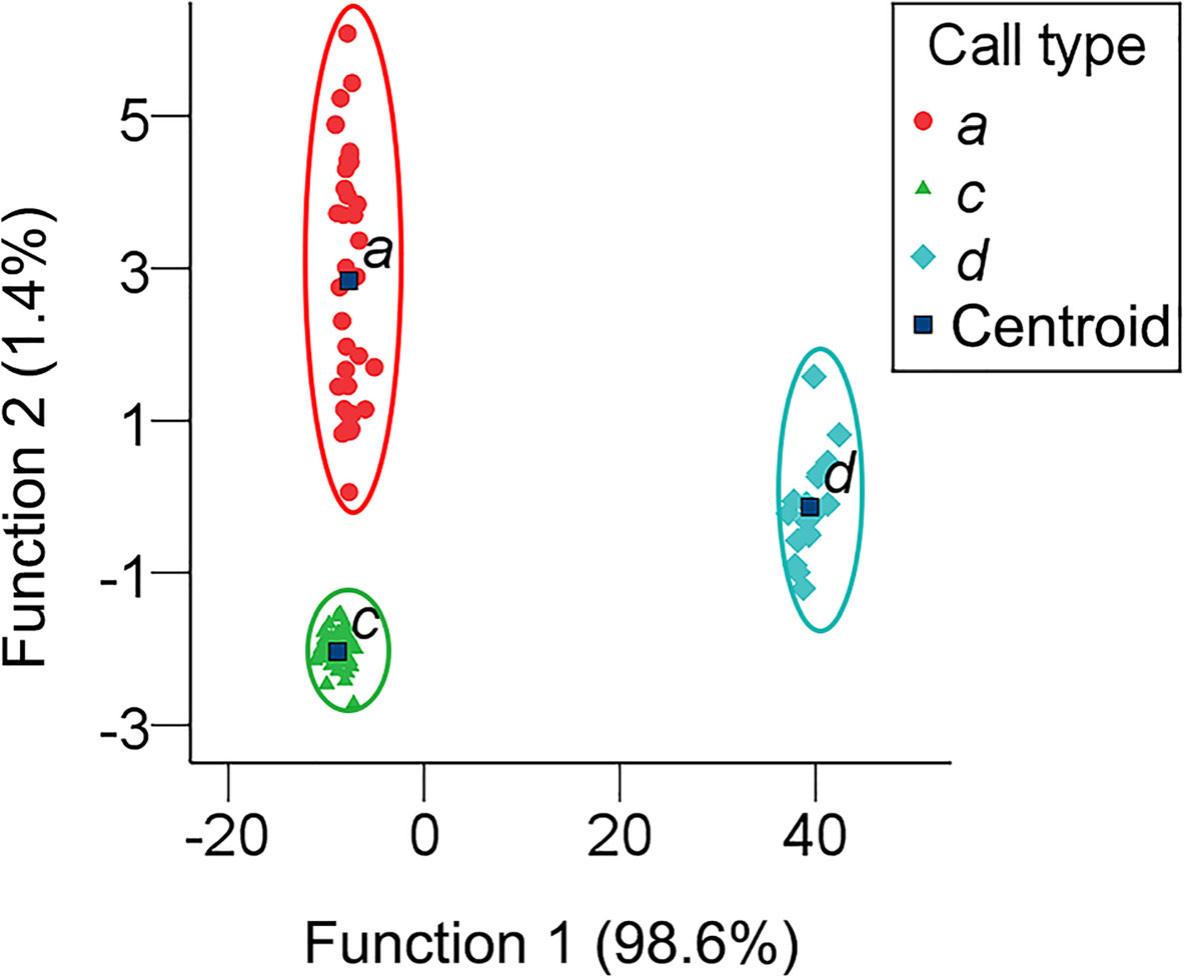
Discriminant function analysis (DFA) indicating that calls *a*, *c*, and *d* can be separated completely by the first 2 discriminant functions

We tested whether the common coot used distinct call modes by examining acoustic structure and context-dependent variation in their vocalizations during the breeding season. First and foremost, Shapiro-Wilk test was conducted to test for normality of all variables, the parameters of all call types were approximated to a normal distribution; thus, one-way analysis of variance (ANOVA) was used to analyze significant differences in the parameters of call types *a*, *c* and *d*, and followed by least-significant difference (LSD) tests for pairwise comparisons because of comparison among 3 samples. The F_0max_ and F_0min_ of call *b* were not measured because the edge of the harmonic was vague. Call *b* was not analyzed because of its small sample size (*n* = 2). Potential discrimination among calls from different contexts was tested using discriminant function analysis (DFA) to classify different call types (*a*, *c* and *d*) by their behavioral context. The DFA (Table 6; Fig. 5) classified them clearly by F_0min_ (explaining 70.0% of the total variance) in function 1 and T (explaining 92.2% of the total variance) in function 2.

**Table 6:** Discriminant function analysis (DFA) of call types *a*, *c*, and *d*. Eigenvalues, percent variance, and the standardized canonical discriminant function coefficients of functions and parameters

Functions	Eigenvalue	Percent variance (%)	Peak frequency (PF, Hz)	Fundamental frequency (F_0_, Hz)	Maximum frequency of F_0_ (F_0max_, Hz)	Minimum frequency of F_0_ (F_0min_, Hz)	Duration of syllable (T, s)	Interval of syllables (TI, s)
Function 1	339.294	98.6	−0.021	0.460	0.389	0.700	−0.009	0.072
Function 2	4.922	1.4	0.121	0.003	0.034	−0.049	0.922	−0.263

**Fig. 6:**
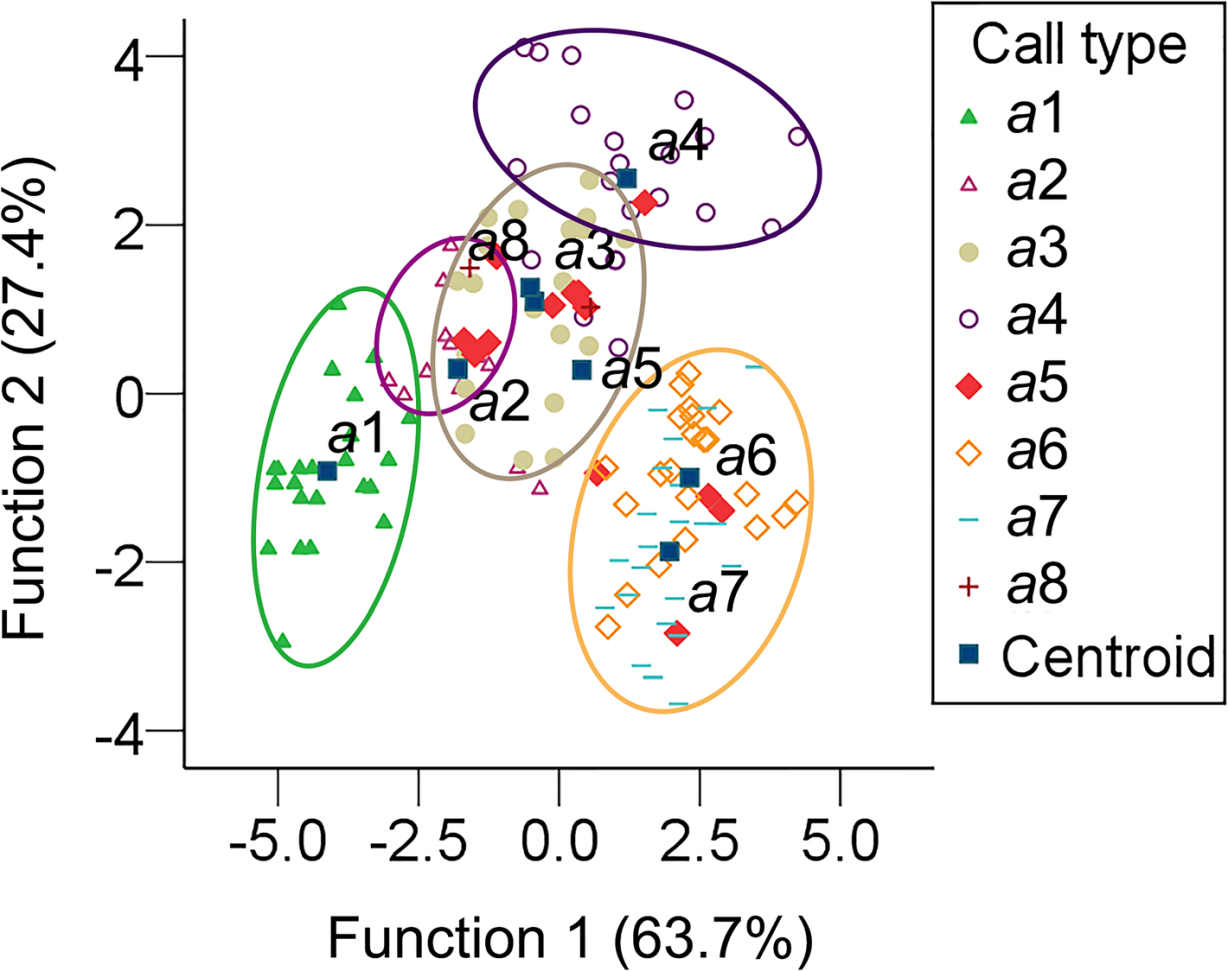
Results of discriminant function analysis (DFA) of different call subtypes of *a* under 8 different behaviors

Among the 4 call types, song type *a* was the most commonly used during various behaviors; thus, one-way analysis of variance was also performed to analyze significant differences in the parameters of call *a*1 and calls *a*3–*a*7; calls *a*2 and *a*8 were not analyzed because of their small sample sizes (*n* = 1). We used the DFA to determine if acoustic variation in song type *a* was associated with different behavioral purposes. According to the results of the DFA, calls *a*1–*a*5 and *a*8 were distinct from *a*6–*a*7 by F_0max_ and TI (explaining 91.6 and 90.3% of the total variance respectively) in function 1, and F_0max_ (explaining 93.8% of the total variance) in function 2 (Table 7; Fig. 6). These statistical analyses were performed in IBM SPSS ver. 23 for Windows (SPSS Inc., Chicago, USA). However, we did not analyze significant differences in the parameters *b*, *c*, and *d* under different behaviors because of the small sample size (*b*8, *b*9, *c*5, *c*6, *d*3, and *d*6, only one individual in some cases but with a few syllables). Finally, we ran a linear mixed model (LMM) with call types (*a*1 and *a*3–*a*7) as the response variable, PF, F_0_, F_0max_, F_0min_ and T (except TI) as fixed effects and individual identity (ID) as random effect using the ‘lmer’ of the ‘lmerTest’ R package [52] in R ver. 4.0.5 (The R Foundation for Statistical Computing, Vienna, Austria, http://www.r-project.org); *a*2 and *a*8 with only one individual were dropped from the model. Because the edge of the harmonic of *b* and *c*5 was vague, we did not measure the F_0max_ and F_0min_ of them. Data were presented as mean ± *SD*. *P* < 0.05 and *P* < 0.01 was regarded as statistically significant and highly significant, respectively.

**Table 7:** Discriminant function analysis (DFA) of calls *a*1–*a*8. Eigenvalues, percent variance, and standardized canonical discriminant function coefficients of functions and parameters

Functions	Eigenvalue	Percent variance (%)	Peak frequency (PF, Hz)	Fundamental frequency (F_0_, Hz)	Maximum frequency of F_0_ (F_0max_, Hz)	Minimum frequency of F_0_ (F_0min_, Hz)	Duration of syllable (T, s)	Interval of syllables (TI, s)
Function 1	5.225	63.7	0.229	−0.436	0.916	−0.711	− 0.608	0.903
Function 2	2.242	27.4	0.554	−0.548	0.938	0.149	0.421	0.017
Function 3	0.632	7.7	0.460	0.906	−0.212	−0.538	−0.003	− 0.168
Function 4	0.088	1.1	−0.376	0.595	−0.180	0.617	−0.164	0.179
Function 5	0.008	0.1	0.593	0.167	−0.787	0.417	0.244	0.469
Function 6	0.002	0.0	−0.312	0.901	0.134	−0.719	0.803	0.397

## Supplementary Information

**Additional file 1 **Table S1. Characteristics of call *a* under 8 different behavioral contexts (*a*1–*a*8) observed in adult common coots. *n* is the number of individuals. Results of a one-way ANOVA revealed significant differences between call *a*1 and calls *a*3–*a*7. Paired comparisons between each of the 2 call types were subjected to least-significant difference tests.

**Additional file 2.** Table S2. Summary of concrete information included in all recordings of vocalizations of adult common coot.

**Additional file 3 **Fig. S1. Spectrograms of all call types of *a*1–*a*8 under 8 behaviors and *b*9 that produced when adults communicate with nestlings.

## Data Availability

The datasets supporting the conclusions of this article are available in the. Figshare repository [10.6084/m9.figshare.15085758.v1].

## Abbreviations

ANOVA: Analysis of variance
DFA: Discriminant function analysis
F_0_: Fundamental frequency (Hz)
FFT: Fast Fourier transform
F_0max_: Maximum frequency of fundamental frequency (Hz)
F_0min_: Minimum frequency of fundamental frequency (Hz)
ID: Individual identity
LMM: Linear mixed model
LSD: Least-significant difference
*n*: Number of individuals
PF: Peak frequency (Hz)
*SD*: Standard deviation
*SE*: Standard error
S/N: Signal-to-noise ratio
T: Duration of syllable (s)
TI: Interval of syllables (s)
