# Supplementary material for: A small vocal repertoire during the breeding season expresses complex behavioral motivations and individual signature in the common coot
Source: BMC Zool. 2021 Sep 2;6:24. doi: 10.1186/s40850-021-00088-4 (PMC10127384; doi:10.1186/s40850-021-00088-4)
Supplement: Supplementary file 1 — Additional file 1 Table S1. Characteristics of call a under 8 different behavioral contexts (a1–a8) observed in adult common coots. n is the number of individuals. Results of a one-way ANOVA revealed significant differences between call a1 and calls a3–a7. Paired comparisons between each of the 2 call types were subjected to least-significant difference tests. [file 40850_2021_88_MOESM1_ESM.docx]

Table S1. Characteristics of call *a* under 8 different behavioral contexts (*a*1–*a*8) observed in adult common coots. *n* is the number of individuals. Results of a one-way ANOVA revealed significant differences between call *a*1 and calls *a*3–*a*7. Paired comparisons between each of the 2 call types were subjected to least-significant difference tests.

| Call type | Behaviors | Peak frequency  (PF, Hz) | Fundamental frequency  (F_0_, Hz) | Maximum frequency of F_0_  (F_0max_, Hz) | Minimum frequency of F_0_ (F_0min_, Hz) | Duration of syllables (T, s) | Interval of syllables (TI, s) |
| --- | --- | --- | --- | --- | --- | --- | --- |
| *a*1 | *courtship* | 1485±615  (*n* = 6) | 900±104  (*n* = 6) | 1251±122  (*n* = 6) | 585±100  (*n* = 6) | 0.089±0.017  (*n* = 6) | 0.518±0.071  (*n* = 2) |
| *a*2 | *copulation* | 1669  (*n* = 1) | 922  (*n* = 1) | 1293  (*n* = 1) | 612  (*n* = 1) | 0.089  (*n* = 1) | 0.627  (*n* = 1) |
| *a*3 | *forage* | 1561 ± 505  (*n* = 7) | 928±103  (*n* = 7) | 1267 ±117  (*n* = 7) | 601±96  (*n* = 7) | 0.091±0.027  (*n* = 7) | 0.497 ±0.319  (*n* = 4) |
| *a*4 | *chase and fight* | 1767±609  (*n* = 9) | 975±91  (*n* = 9) | 1380±118  (*n* = 9) | 626±80  (*n* = 9) | 0.081±0.020  (*n* = 9) | 1.112±0.482  (*n* = 2) |
| *a*5 | *back to nest* | 1829±287  (*n* = 2) | 957±88  (*n* = 2) | 1300±102  (*n* = 2) | 599±92  (*n* = 2) | 0.076±0.028  (*n* = 2) | 0.849±0.264  (*n* = 2) |
| *a*6 | *in the nest* | 1252±378  (*n* = 3) | 871±59  (*n* = 3) | 1225±46  (*n* = 3) | 539±42  (*n* = 3) | 0.053±0.009  (*n* = 3) | 1.299±0.184  (*n* = 3) |
| *a*7 | *searching nest materials* | 1200±87  (*n* = 2) | 904±20  (*n* = 2) | 1214±90  (*n* = 2) | 537±62  (*n* = 2) | 0.052±0.007  (*n* = 2) | 1.228±0.045  (*n* = 2) |
| *a*8 | *leaving nest* | 1965  (*n* = 1) | 980  (*n* = 1) | 1355  (*n* = 1) | 650  (*n* = 1) | 0.078  (*n* = 1) | 0.731  (*n* = 1) |
| ANOVA |  |  |  |  |  |  |  |
| *F* |  | 13.956 | 15.919 | 33.020 | 13.394 | 59.485 | 419.656 |
| *P* |  | 0.000** | 0.000** | 0.000** | 0.000** | 0.000** | 0.000** |
| Paired comparisons | |  |  |  |  |  |  |
| *a*1-*a*3 |  | 0.276 | 0.024* | 0.250 | 0.156 | 0.575 | 0.495 |
| *a*1-*a*4 |  | 0.000** | 0.000** | 0.000** | 0.000** | 0.001** | 0.000** |
| *a*1-*a*5 |  | 0.014* | 0.018* | 0.065 | 0.526 | 0.011* | 0.000** |
| *a*1-*a*6 |  | 0.004** | 0.029* | 0.090 | 0.000** | 0.000** | 0.000** |
| *a*1-*a*7 |  | 0.002** | 0.739 | 0.762 | 0.049* | 0.000** | 0.000** |
| *a*3-*a*4 |  | 0.005** | 0.000** | 0.000** | 0.023* | 0.000** | 0.000** |
| *a*3-*a*5 |  | 0.063 | 0.241 | 0.221 | 0.915 | 0.005** | 0.000** |
| *a*3-*a*6 |  | 0.000** | 0.000** | 0.009** | 0.000** | 0.000** | 0.000** |
| *a*3-*a*7 |  | 0.000** | 0.159 | 0.241 | 0.003** | 0.000** | 0.000** |
| *a*4-*a*5 |  | 0.658 | 0.455 | 0.003** | 0.198 | 0.349 | 0.000** |
| *a*4-*a*6 |  | 0.000** | 0.000** | 0.000** | 0.000** | 0.000** | 0.000** |
| *a*4-*a*7 |  | 0.000** | 0.000** | 0.000** | 0.000** | 0.000** | 0.000** |
| *a*5-*a*6 |  | 0.000** | 0.001** | 0.007** | 0.006** | 0.000** | 0.000** |
| *a*5-*a*7 |  | 0.000** | 0.049* | 0.061 | 0.077 | 0.000** | 0.000** |
| *a*6-*a*7 |  | 0.554 | 0.043* | 0.292 | 0.197 | 0.747 | 0.013* |

* *P* < 0.05 ** *P* < 0.01
