# Supplementary material for: A small vocal repertoire during the breeding season expresses complex behavioral motivations and individual signature in the common coot
Source: BMC Zool. 2021 Sep 2;6:24. doi: 10.1186/s40850-021-00088-4 (PMC10127384; doi:10.1186/s40850-021-00088-4)
Supplement: Supplementary file 2 — Additional file 2. Table S2. Summary of concrete information included in all recordings of vocalizations of adult common coot. [file 40850_2021_88_MOESM2_ESM.docx]

Table S2. Summary of concrete information included in all recordings of vocalizations of adult common coot.

| Recording | Call type | Behavioral contexts | Number of calls for analysis | Number of individuals | Individual identity | Date of recording (year, month, day) |
| --- | --- | --- | --- | --- | --- | --- |
| 1 | *a*1 | *Courtship* | 38 | 2 | No.1, No.2 | 20080509 |
| 2 | *a*1 | *Courtship* | 8 | 2 | No.1, No.2 | 20080509 |
| 3 | *a*1 | *Courtship* | 11 | 2 | No.1, No.2 | 20080509 |
| 4 | *a*1 | *Courtship* | 25 | 1 | No.3 | 20080511 |
| 5 | *a*1 | *Courtship* | 4 | 1 | No.3 | 20080511 |
| 6 | *a*1 | *Courtship* | 6 | 1 | No.3 | 20080511 |
| 7 | *a*1 | *Courtship* | 20 | 2 | No.4, No.5 | 20080510 |
| 8 | *a*1 | *Courtship* | 11 | 2 | No.3, No.6 | 20080511 |
| 9 | *a*2 | *Copulation* | 39 | 1 | No.3 | 20080510 |
| 10 | *a*3 | *Forage* | 9 | 1 | No.7 | 20080511 |
| 11 | *a*3 | *Forage* | 8 | 1 | No.8 | 20080514 |
| 12 | *a*3 | *Forage* | 7 | 1 | No.9 | 20080514 |
| 13 | *a*3 | *Forage* | 18 | 1 | No.10 | 20080424 |
| 14 | *a*3 | *Forage* | 6 | 1 | No.10 | 20080424 |
| 15 | *a*3 | *Forage* | 2 | 1 | No.11 | 20080510 |
| 16 | *a*3 | *Forage* | 2 | 1 | No.12 | 20080509 |
| 17 | *a*3 | *Forage* | 2 | 1 | No.12 | 20080509 |
| 18 | *a*3 | *Forage* | 8 | 1 | No.11 | 20080510 |
| 19 | *a*3 | *Forage* | 9 | 1 | No.8 | 20080514 |
| 20 | *a*3 | *Forage* | 2 | 1 | No.13 | 20080511 |
| 21 | *a*3 | *Forage* | 3 | 1 | No.3 | 20080513 |
| 22 | *a*3 | *Forage* | 1 | 1 | No.3 | 20080513 |
| 23 | *a*3 | *Forage* | 3 | 1 | No.8 | 20080514 |
| 24 | *a*3 | *Forage* | 4 | 1 | No.8 | 20080514 |
| 25 | *a*3 | *Forage* | 4 | 1 | No.3 | 20080513 |
| 26 | *a*4 | *Chase and fight* | 15 | 1 | No.3 | 20080510 |
| 27 | *a*4 | *Chase and fight* | 7 | 1 | No.14 | 20080510 |
| 28 | *a*4 | *Chase and fight* | 2 | 1 | No.14 | 20080510 |
| 29 | *a*4 | *Chase and fight* | 19 | 1 | No.15 | 20080423 |
| 30 | *a*4 | *Chase and fight* | 34 | 2 | No.14, No.16 | 20080519 |
| 31 | *a*4 | *Chase and fight* | 15 | 2 | No.17, No.18 | 20080511 |
| 32 | *a*4 | *Chase and fight* | 14 | 2 | No.19, No.20 | 20080511 |
| 33 | *a*4 | *Chase and fight* | 25 | 1 | No.21 | 20080514 |
| 34 | *a*5 | *Back to nest* | 4 | 1 | No.3 | 20080513 |
| 35 | *a*5 | *Back to nest* | 1 | 1 | No.3 | 20080513 |
| 36 | *a*5 | *Back to nest* | 8 | 1 | No.3 | 20080511 |
| 37 | *a*5 | *Back to nest* | 3 | 1 | No.19 | 20080511 |
| 38 | *a*6 | *In the nest* | 16 | 1 | No.3 | 20080511 |
| 39 | *a*6 | *In the nest* | 11 | 1 | No.6 | 20080510 |
| 40 | *a*6 | *In the nest* | 12 | 1 | No.3 | 20080513 |
| 41 | *a*6 | *In the nest* | 30 | 1 | No.22 | 20080513 |
| 42 | *a*6 | *In the nest* | 3 | 1 | No.3 | 20080511 |
| 43 | *a*6 | *In the nest* | 1 | 1 | No.3 | 20080511 |
| 44 | *a*7 | *Searching nest materials* | 20 | 1 | No.3 | 20080513 |
| 45 | *a*7 | *Searching nest materials* | 25 | 1 | No.23 | 20080511 |
| 46 | *a*8 | *Leaving nest* | 2 | 1 | No.3 | 20080513 |
| 47 | *b*8 | *Leaving nest* | 7 | 1 | No.3 | 20080511 |
| 48 | *b*9 | *Communication with nestlings* | 208 | 1 | No.24 | 20080617 |
| 49 | *c*5 | *Back to nest* | 4 | 1 | No.25 | 20080511 |
| 50 | *c*6 | *In the nest* | 2 | 1 | No.3 | 20080510 |
| 51 | *c*6 | *In the nest* | 6 | 1 | No.3 | 20080510 |
| 52 | *c*6 | *In the nest* | 2 | 1 | No.3 | 20080510 |
| 53 | *c*6 | *In the nest* | 26 | 1 | No.3 | 20080511 |
| 54 | *c*6 | *In the nest* | 9 | 1 | No.26 | 20080511 |
| 55 | *c*6 | *In the nest* | 5 | 1 | No.3 | 20080511 |
| 56 | *c*6 | *In the nest* | 5 | 1 | No.27 | 20080514 |
| 57 | *d*3 | *Forage* | 2 | 1 | No.28 | 20080514 |
| 58 | *d*3 | *Forage* | 2 | 1 | No.29 | 20080514 |
| 59 | *d*3 | *Forage* | 5 | 1 | No.30 | 20080514 |
| 60 | *d*6 | *In the nest* | 6 | 1 | No.26 | 20080511 |
| 61 | *d*6 | *In the nest* | 3 | 1 | No.26 | 20080511 |
