# Supplementary material for: A small vocal repertoire during the breeding season expresses complex behavioral motivations and individual signature in the common coot
Source: BMC Zool. 2021 Sep 2;6:24. doi: 10.1186/s40850-021-00088-4 (PMC10127384; doi:10.1186/s40850-021-00088-4)
Supplement: Supplementary file 3 — Additional file 3 Fig. S1. Spectrograms of all call types of a1–a8 under 8 behaviors and b9 that produced when adults communicate with nestlings. [file 40850_2021_88_MOESM3_ESM.docx]

Additional file 3


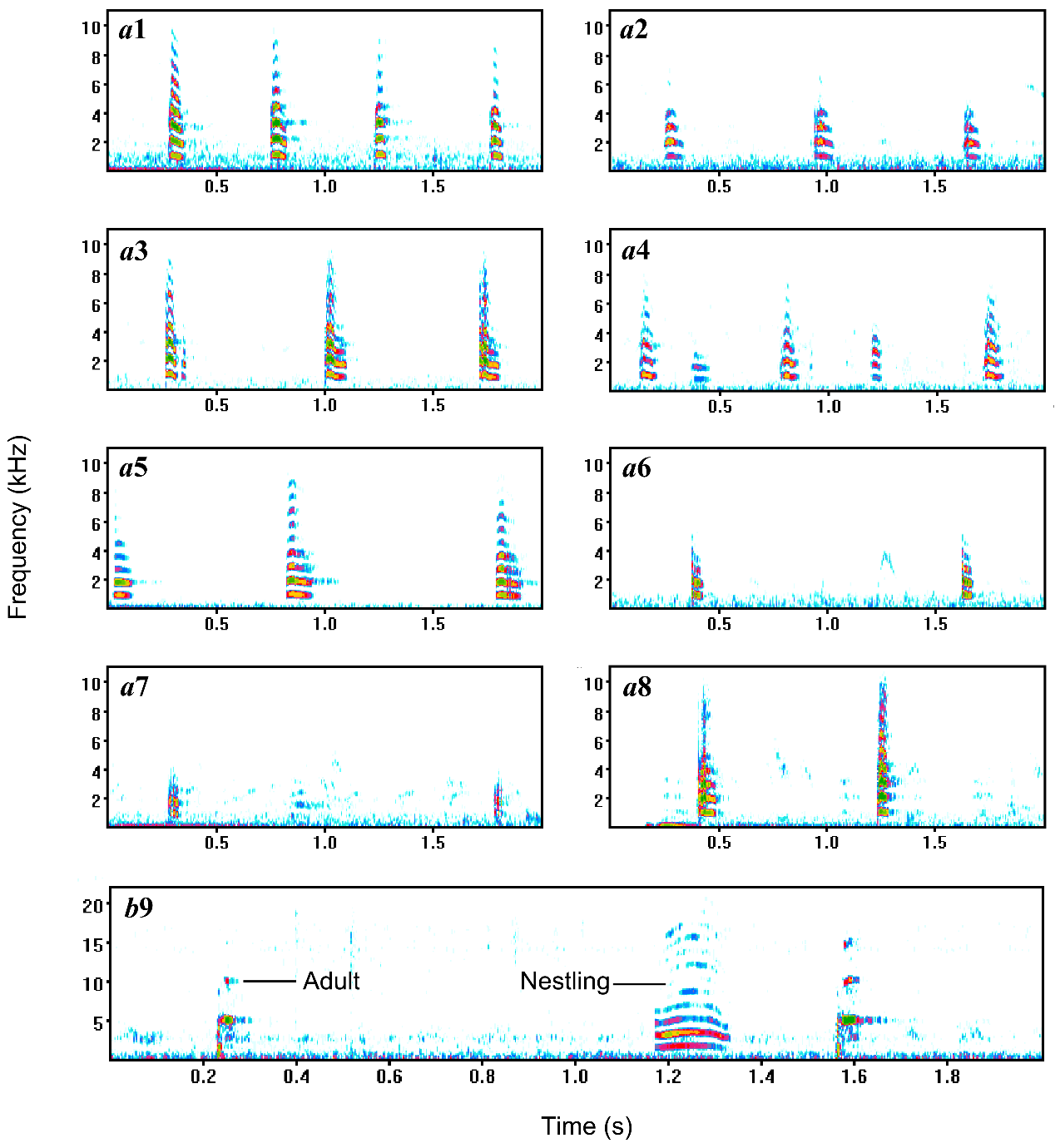


Figure S1. Spectrograms of all call types of *a*1–*a*8 under 8 behaviors and *b*9 that produced when adults communicate with nestlings.
